# Supplementary material for: The deep conservation of the Lepidoptera Z chromosome suggests a non-canonical origin of the W
Source: Nat Commun. 2017 Nov 14;8:1486. doi: 10.1038/s41467-017-01663-5 (PMC5684275; doi:10.1038/s41467-017-01663-5)
Supplement: Supplementary file 1 — Supplementary Information [file 41467_2017_1663_MOESM1_ESM.pdf]

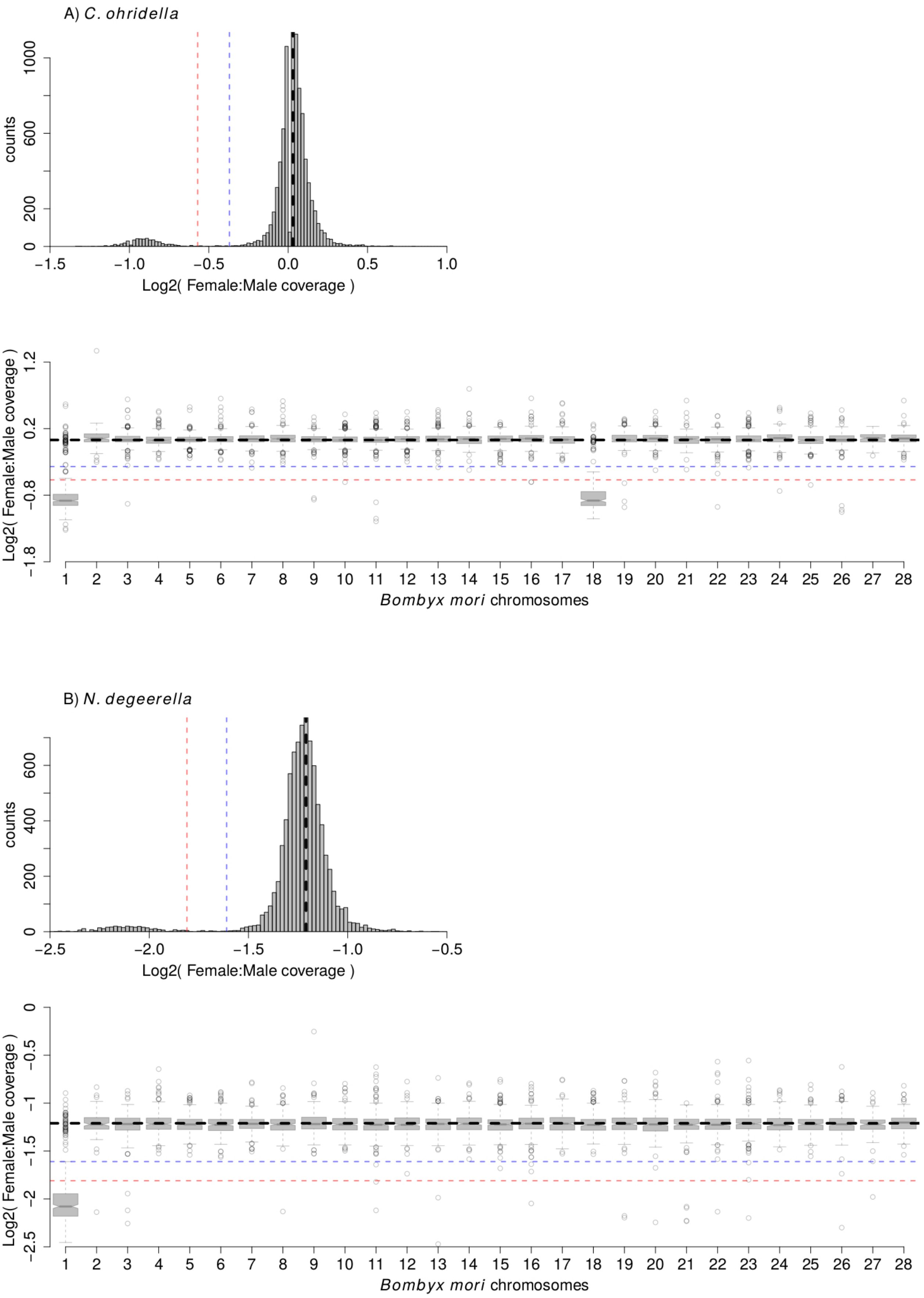
A) C. ohridella
counts
0
200
600
1000
−1.5
−1.0
−0.5
0.0
0.5
1.0
Log2(Female:Male coverage)
Log2(Female:Male coverage)
1.2
0.2
−0.8
−1.8
1 2 3 4 5 6 7 8 9 10 11 12 13 14 15 16 17 18 19 20 21 22 23 24 25 26 27 28
Bombyx mori chromosomes
B) N. degeerella
counts
0
200
400
600
−2.5
−2.0
−1.5
−1.0
−0.5
Log2(Female:Male coverage)
Log2(Female:Male coverage)
0
−0.5
−1
−1.5
−2
−2.5
1 2 3 4 5 6 7 8 9 10 11 12 13 14 15 16 17 18 19 20 21 22 23 24 25 26 27 28
Bombyx mori chromosomes

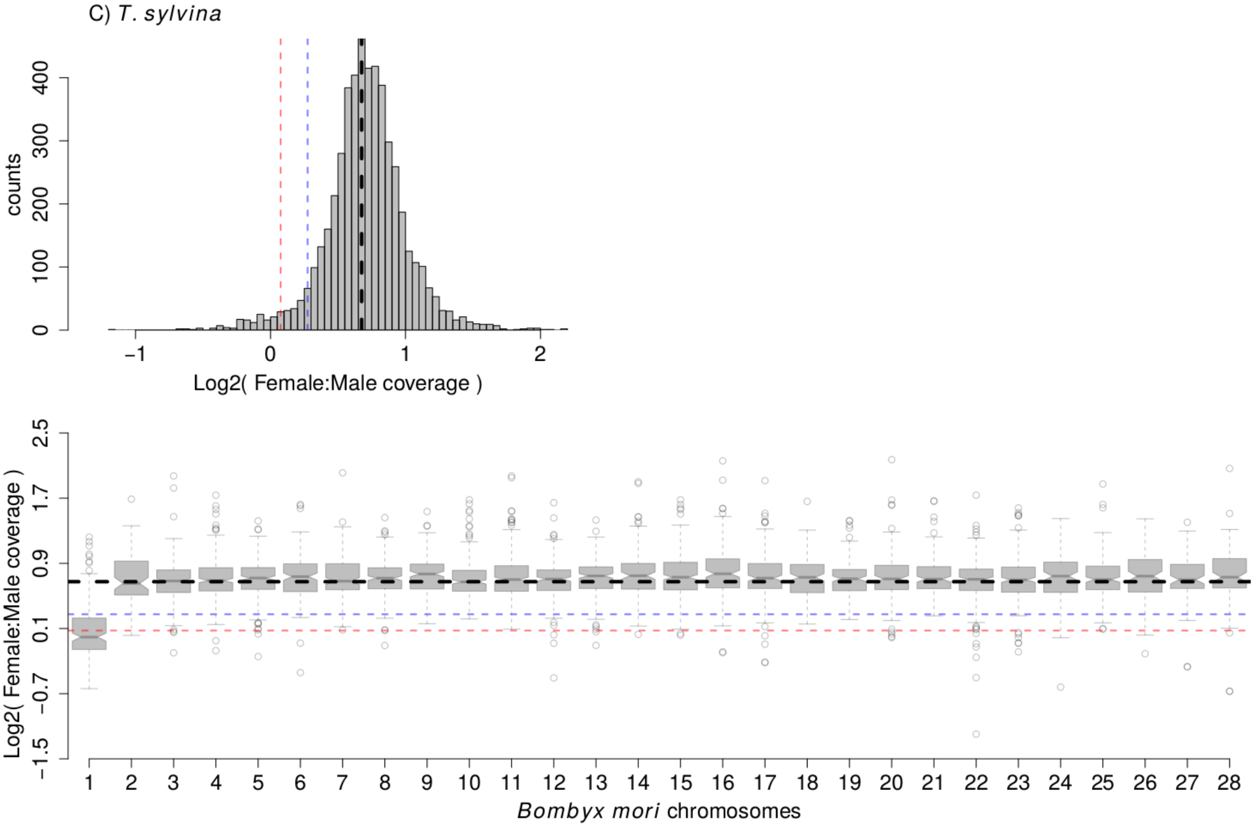

Supplementary Figure 1: The lenient classification is shown for (A) *C. ohridella.* (B) *N. degeerella.* (C) *T. sylvina*. For each species, scaffolds were assigned to one of the *B. mori* chromosomes based on their gene content. The Log2 of the female to male coverage ratio is shown as a whole (top panel), and for each *B. mori* chromosome (low panel). In both panels, the black dashed lines correspond to the autosomal mode “A_mode”, the red dashed lines to the Z-linked cut-off (“A_mode – 0.6”), and the blue dashed lines to the autosomal cut-off (“A_mode – 0.4”).

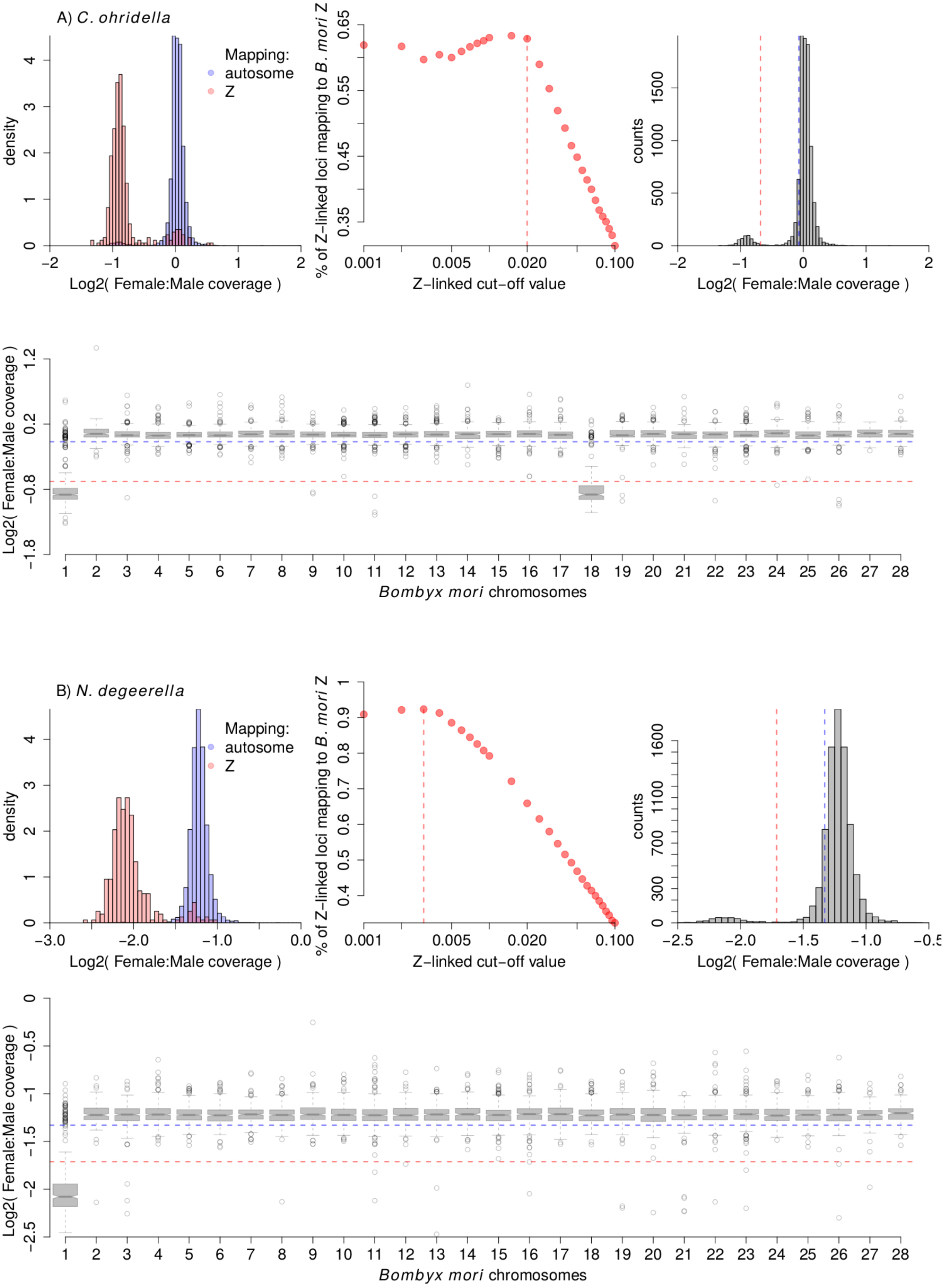

A) C. ohridella
Mapping:
autosome
Z
density
Log2(Female:Male coverage)
% of Z−linked loci mapping to B. mori Z
Z−linked cut−off value
counts
Log2(Female:Male coverage)
Bombyx mori chromosomes
B) N. degeerella
Mapping:
autosome
Z
density
Log2(Female:Male coverage)
% of Z−linked loci mapping to B. mori Z
Z−linked cut−off value
counts
Log2(Female:Male coverage)
Log2(Female:Male coverage)
Bombyx mori chromosomes

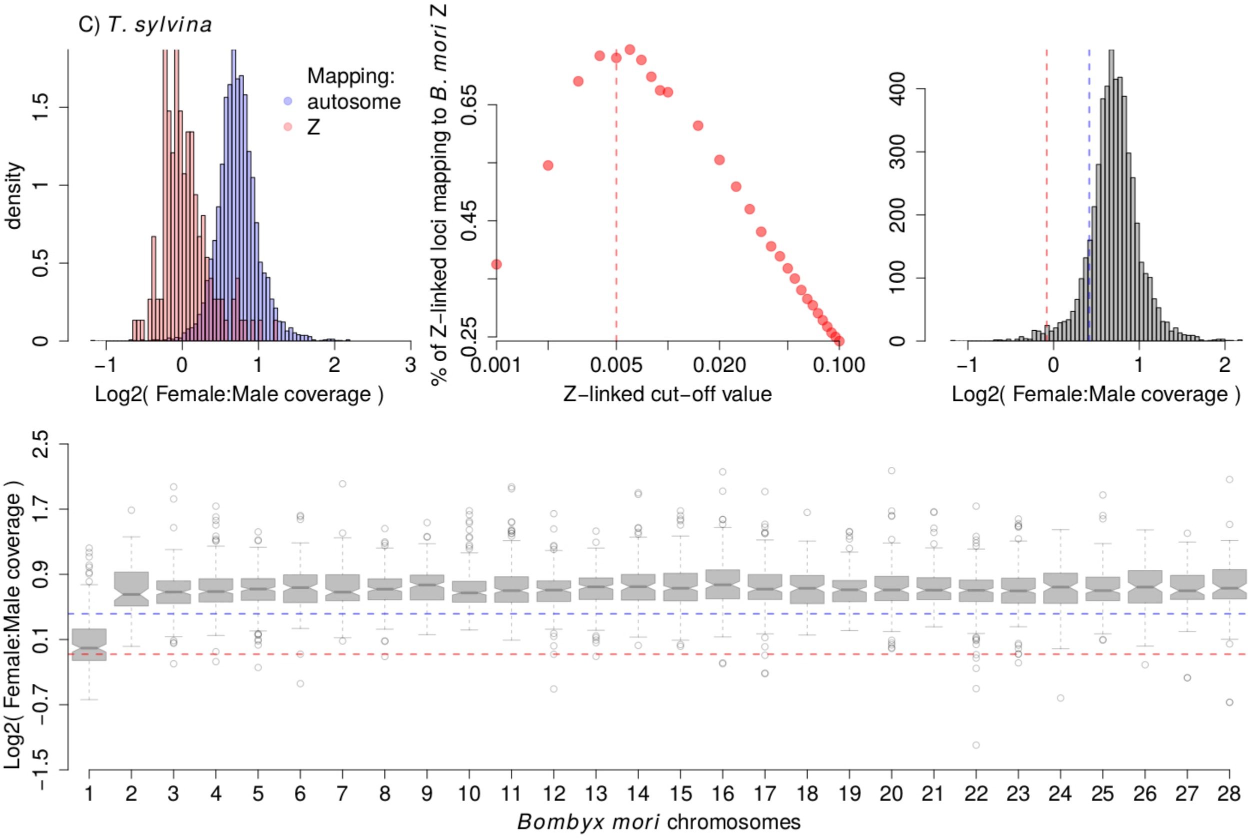

Supplementary Figure 2: The stringent classification is shown for (A) *C. ohridella.* (B) *N. degeerella.* (C) *T. sylvina.* For each species, scaffolds were assigned to one of the *B. mori* chromosomes based on their gene content. In the top leftmost panel, the Log2 of the female to male coverage ratio Log2(F/M coverage) is shown for scaffolds homologous to the *B. mori* Z chromosome (Chr. 1, in red) and scaffolds homologous to *B. mori* autosomes (in blue). The top middle panel shows the proportion of the scaffolds classified as Z-linked which also map to the Z chromosome of *B. mori* as a function of the cut-off value used for the classification. The final Z-linked cut-off value (red dashed line) was chosen as the Log2(F/M coverage) at which this proportion reaches a plateau. The cut-off value is shown on the x-axis as the corresponding percentile of the blue distribution (top leftmost panel). The autosomal cut-off (blue dashed line) was set to the 10th percentile of that blue distribution. The Log2(F/M coverage) is shown as a whole (top rightmost panel), and for each *B. mori* chromosome (low panel). In both panels, the red dashed lines correspond to the Z-linked cut-off and the blue dashed lines to the autosomal cut-off.

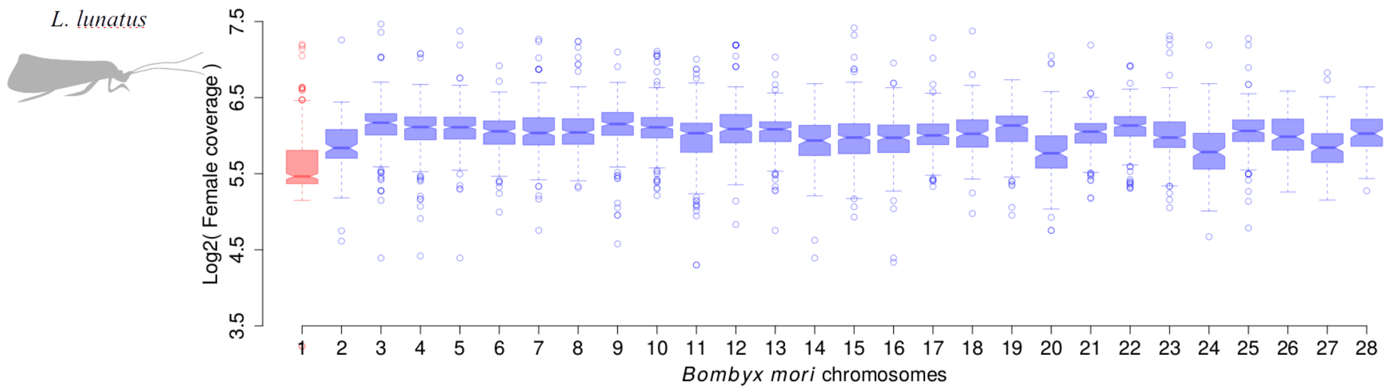

Supplementary Figure 3: The Z chromosome of *B. mori* (Chr. 1) is homologous to the Z chromosome of the sister clade, Trichoptera. The Log2 of the female coverage of each chromosome is shown in *L. lunatus*. All details match Figure 1.

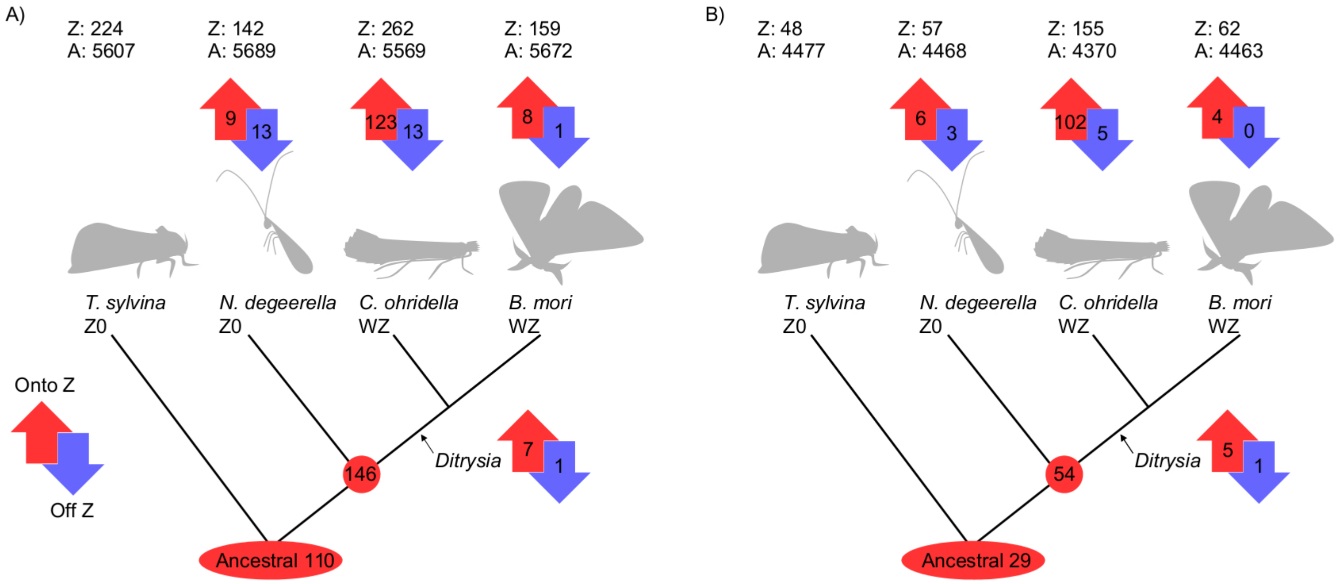

Supplementary Figure 4: Gene movement onto and off the Z chromosome in Lepidoptera obtained by applying a minimal scaffold length of 500 bp. (A) Lenient classification (5,831 genes classified). (B) Stringent classification (4,525 genes classified). The phylogenetic tree is adapted from Regier *et al*[1]. Red circles show the number of genes present on the ancestral Z chromosome. Arrows indicate the number of genes that move onto (up red arrows) or off (down blue arrows) the Z chromosome in each lineage after the split with *T. sylvina*. The top row indicates the total number of genes classified as Z-linked (Z) or autosomal (A) in each species.

A) *C. ohridella*

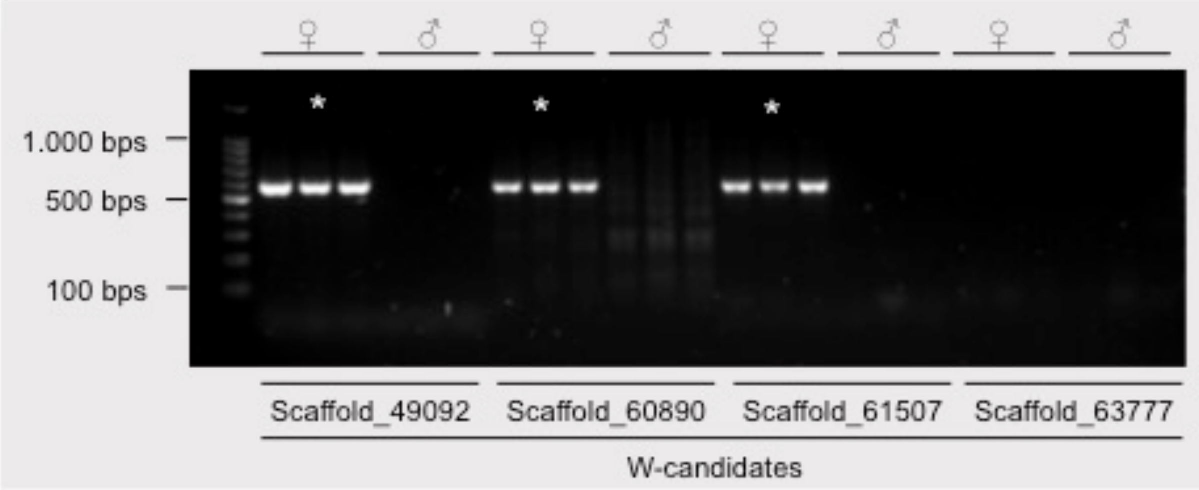

Full gel corresponding to Figure 5B – W candidates

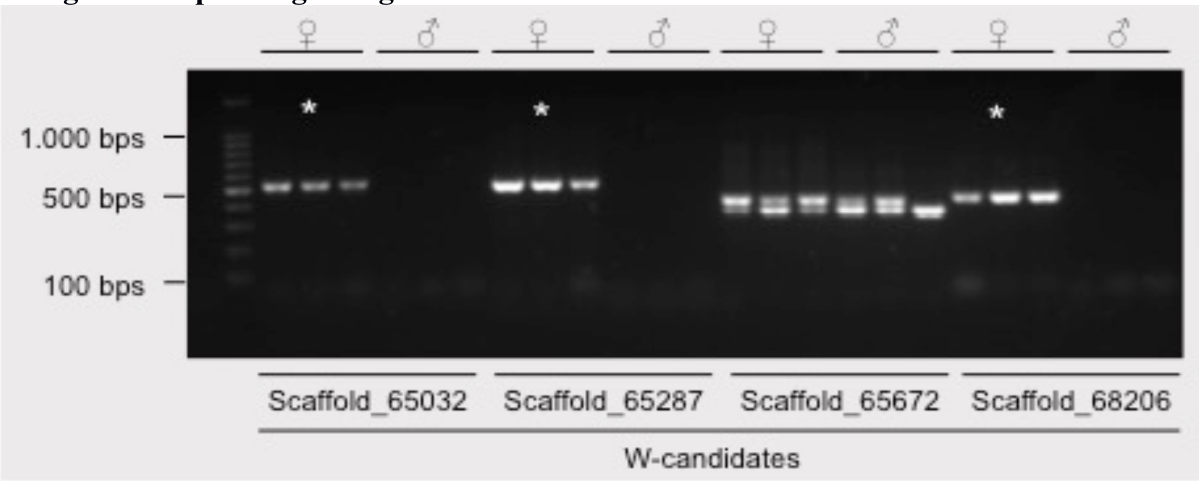

Full gel corresponding to Figure 5A – Autosomal controls

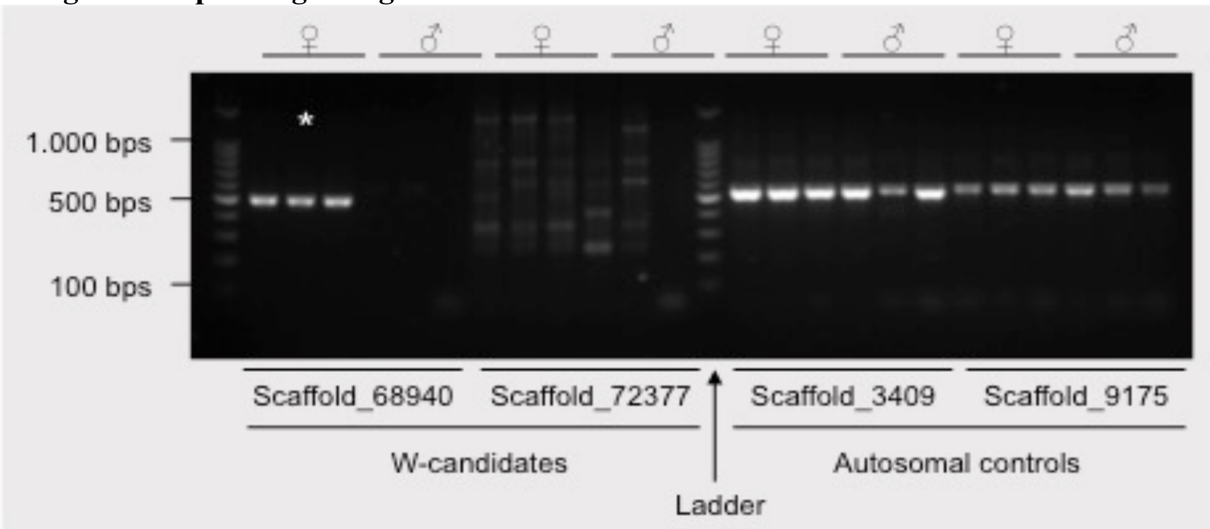

** Asterisks show validated female-specific sequences.*

B) ***N. degeerella***

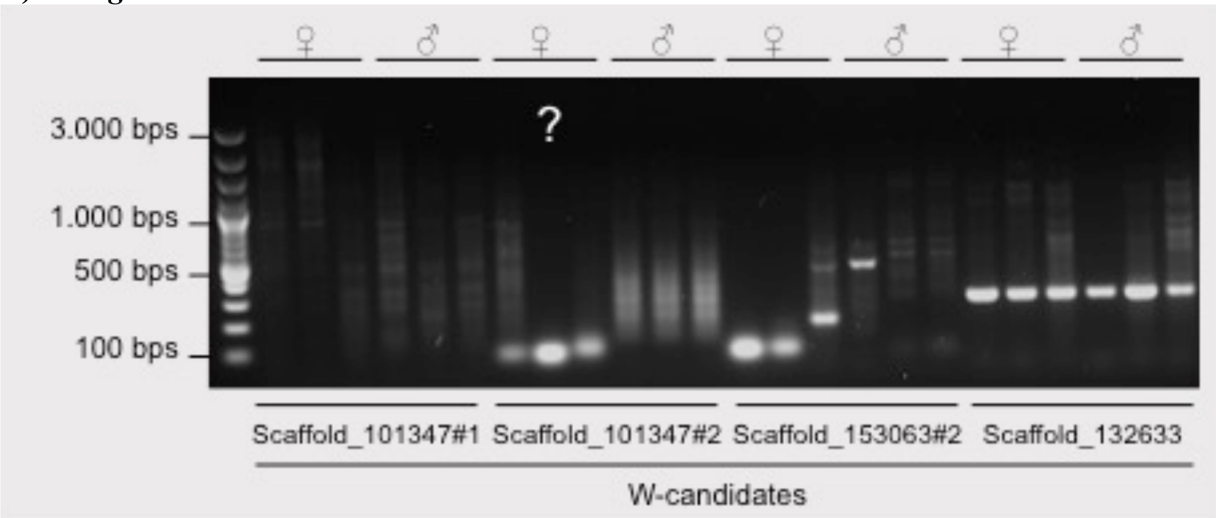

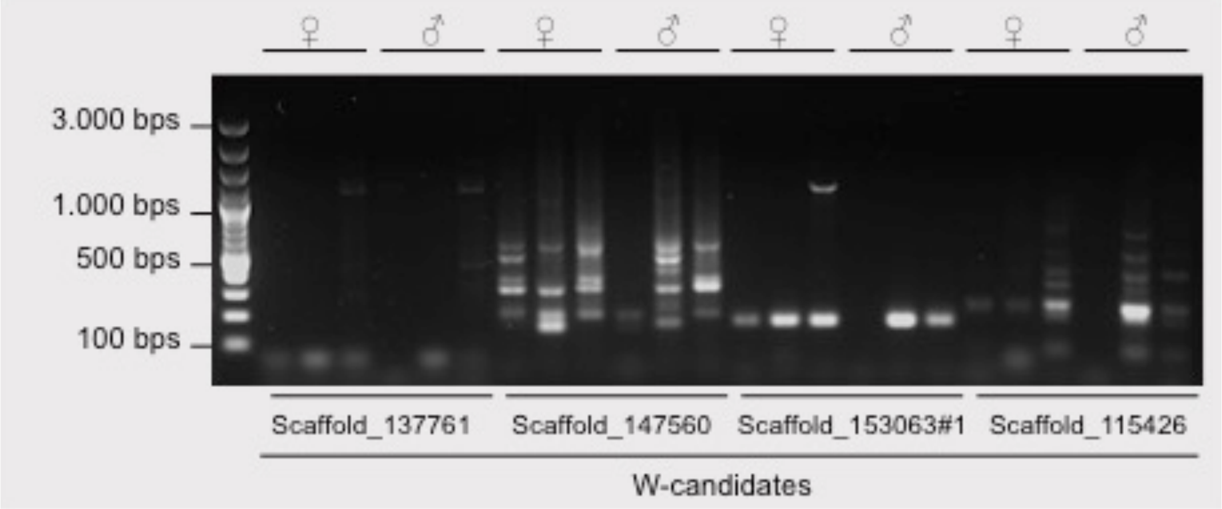

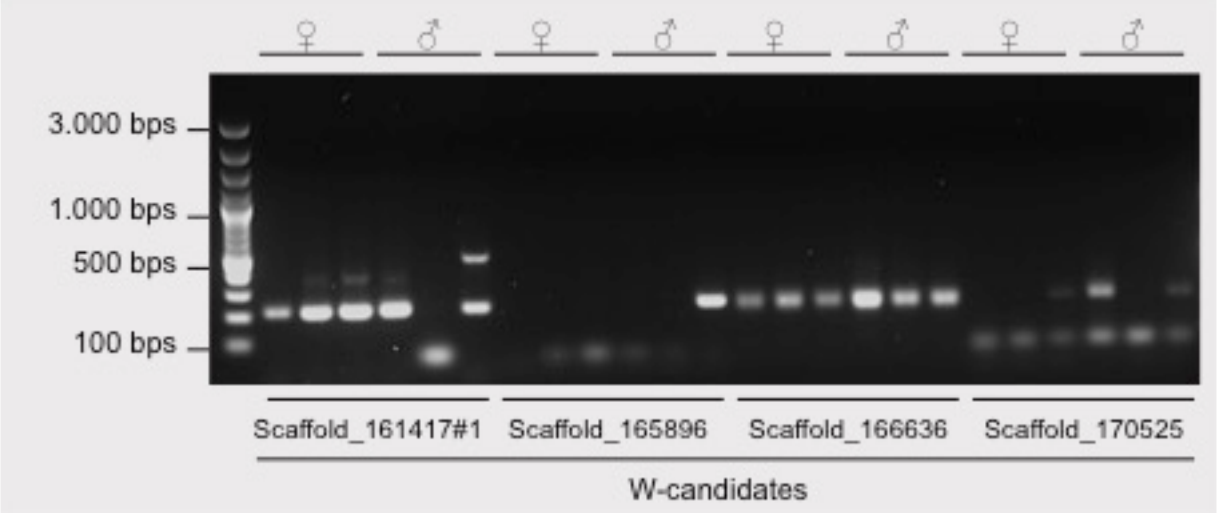

** Question mark shows female specific bands for the Scaffold_101347, using the second pair of primers (#2). It is further tested in new individuals.*

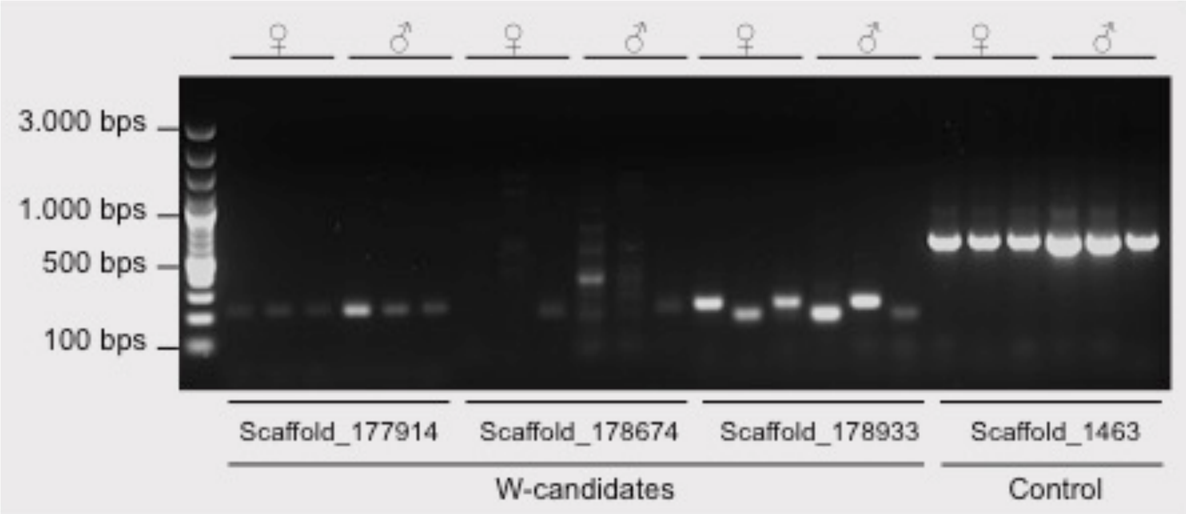
♀ ♂ ♀ ♂ ♀ ♂ ♀ ♂
3.000 bps
1.000 bps
500 bps
100 bps
Scaffold_177914
Scaffold_178674
Scaffold_178933
Scaffold_1463
W-candidates
Control

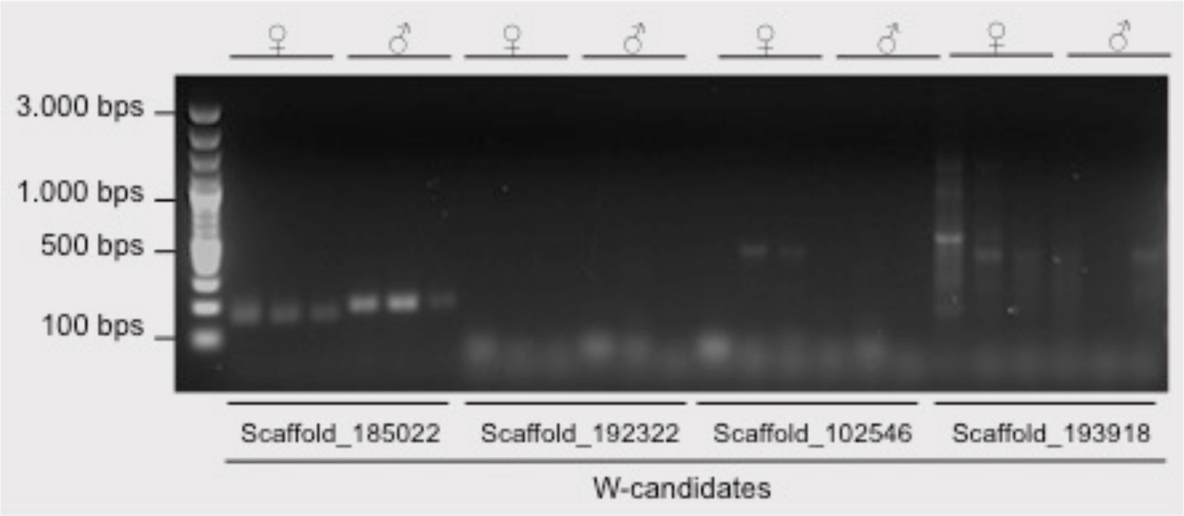
♀ ♂ ♀ ♂ ♀ ♂ ♀ ♂
3.000 bps
1.000 bps
500 bps
100 bps
Scaffold_185022
Scaffold_192322
Scaffold_102546
Scaffold_193918
W-candidates

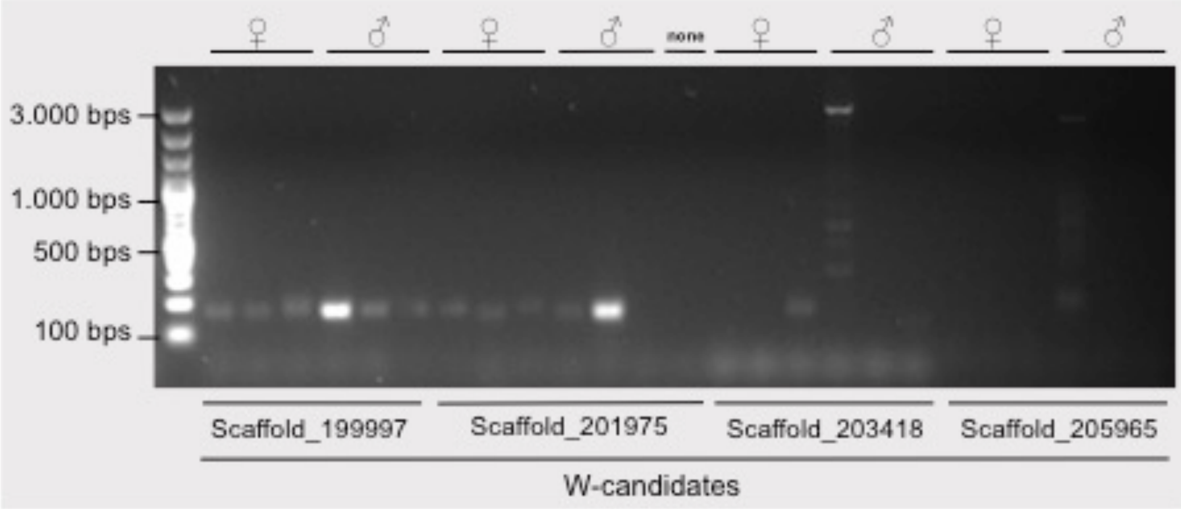
♀ ♂ ♀ ♂ none ♀ ♂ ♀ ♂
3.000 bps
1.000 bps
500 bps
100 bps
Scaffold_199997
Scaffold_201975
Scaffold_203418
Scaffold_205965
W-candidates

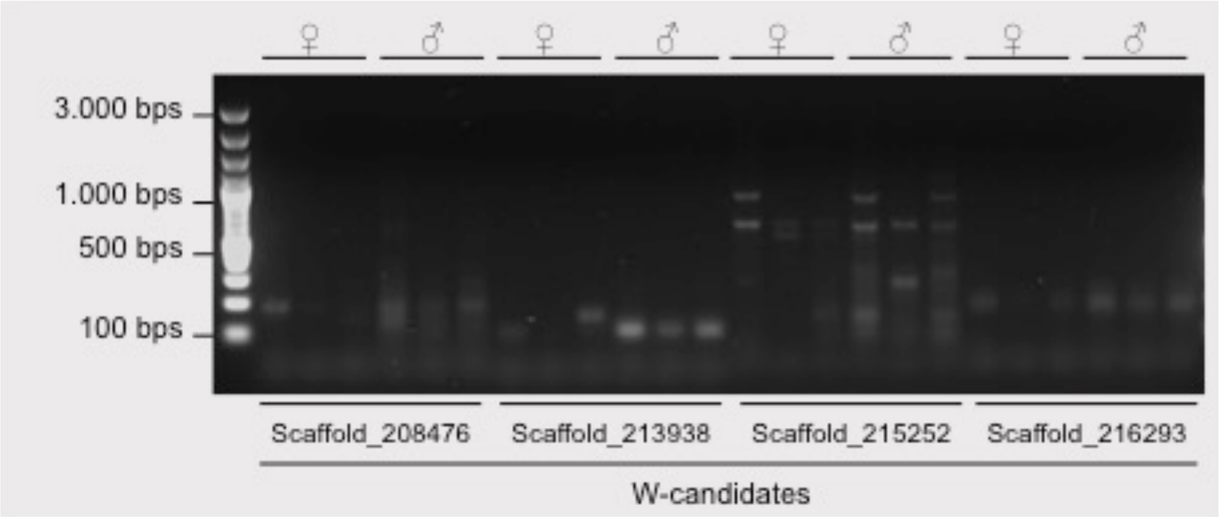

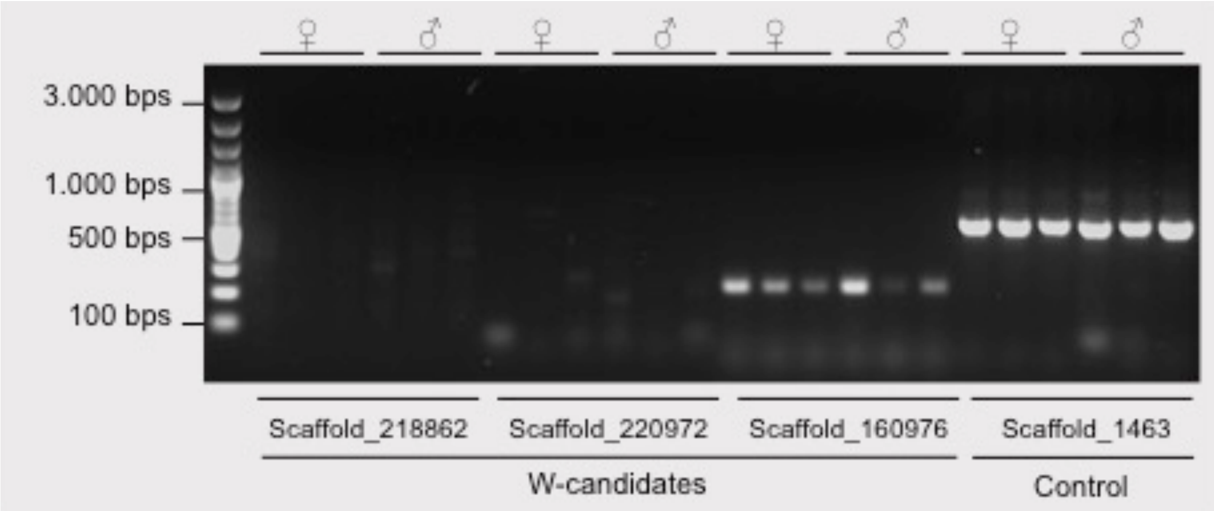

Additional test of W-candidates

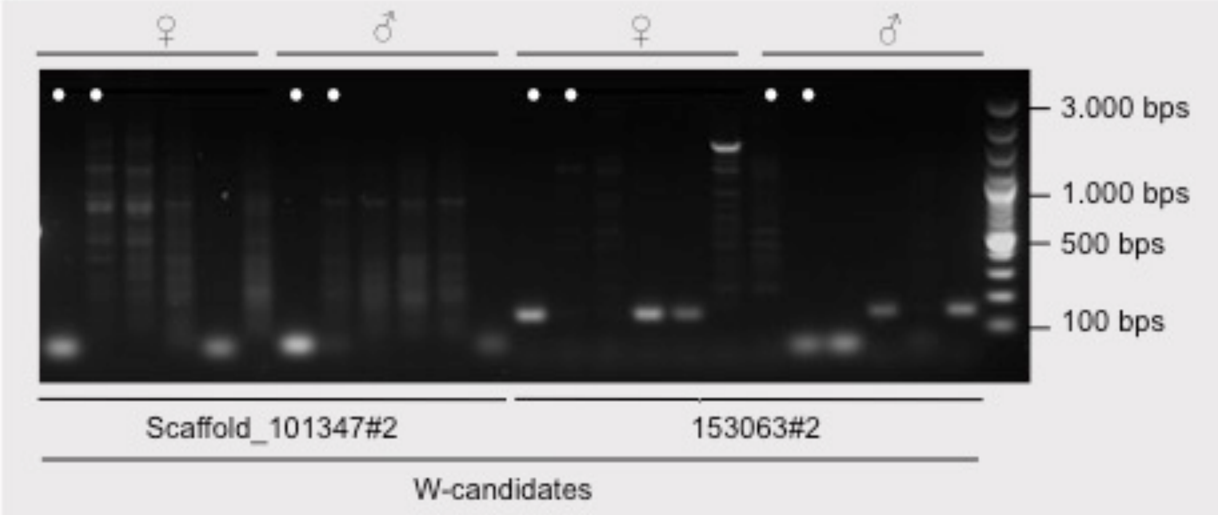

** White spots show two of the three females and two of the three males previously tested. Others are newly tested individuals (four for each sex).*

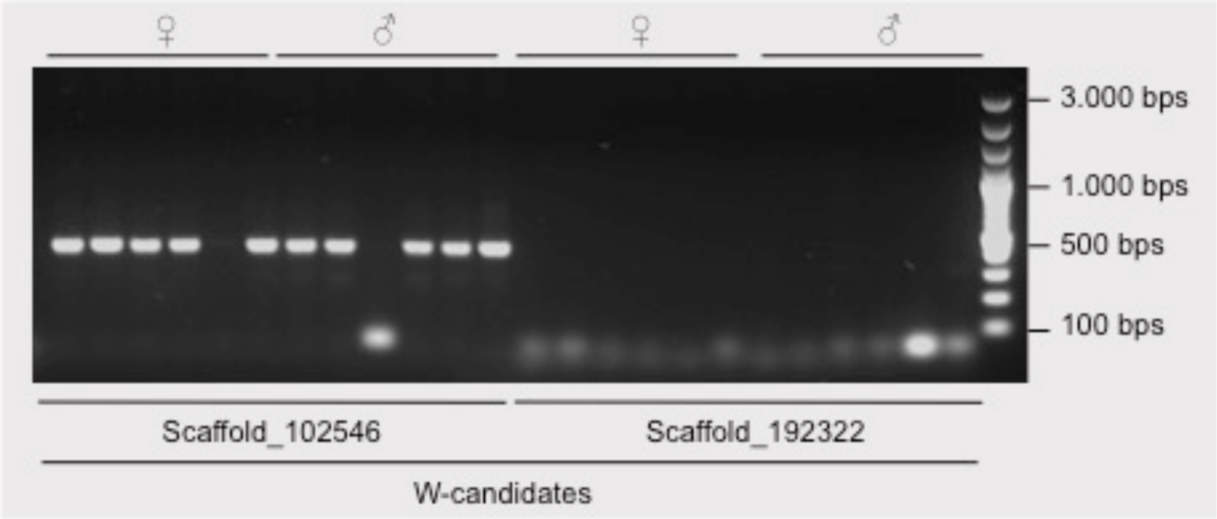

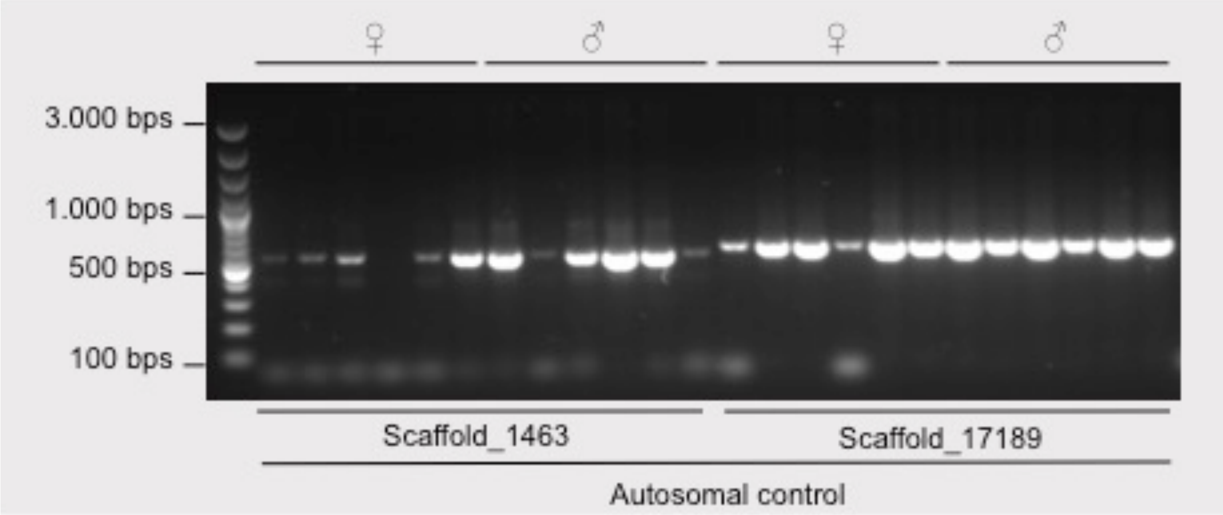

Full gel corresponding to Figure 5B

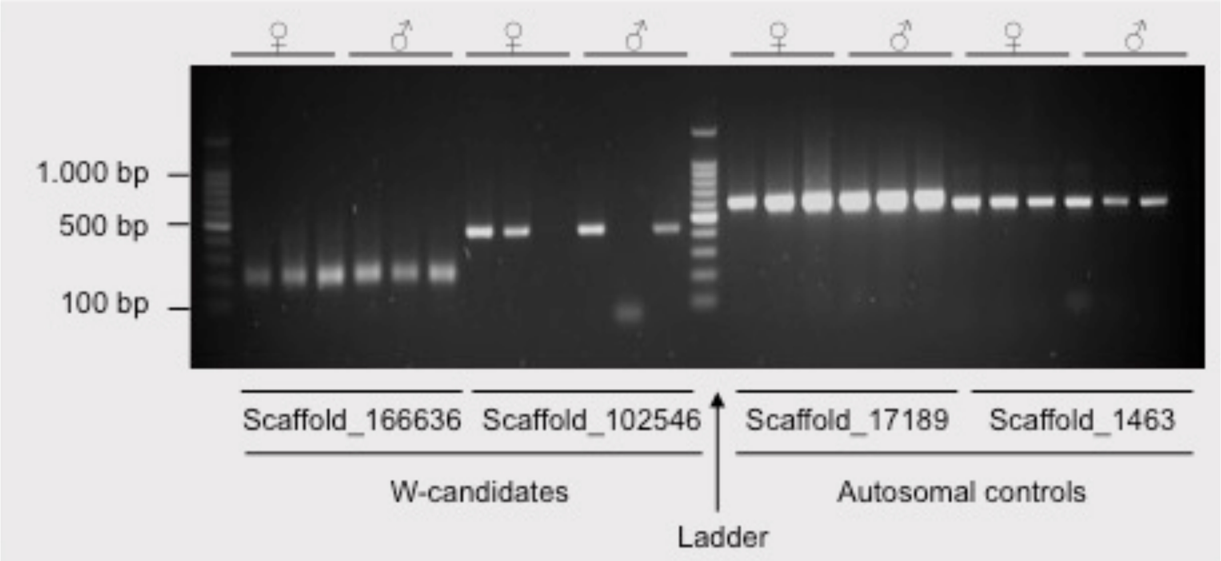

C) ***T. sylvina***

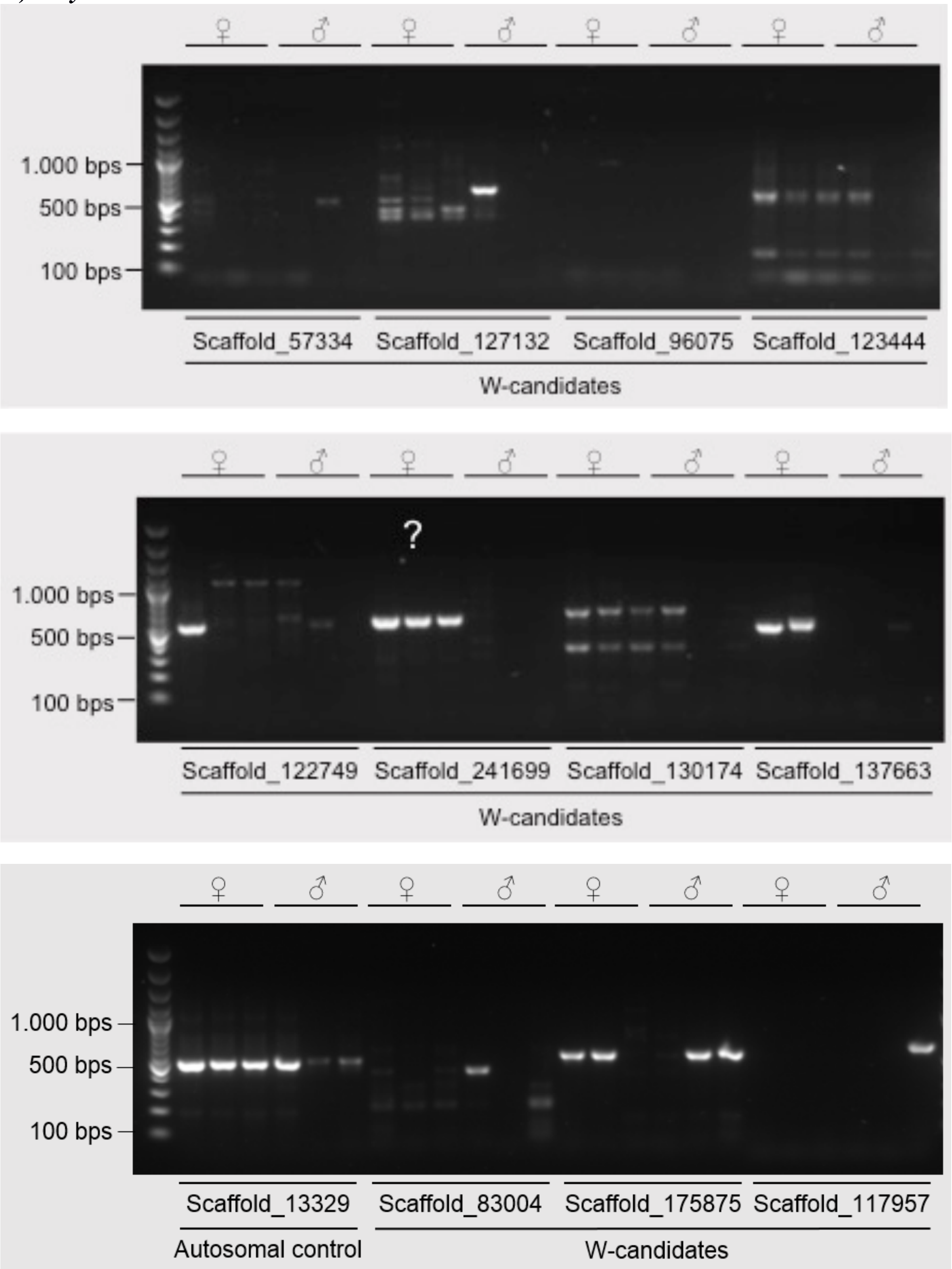

* *Question mark shows female specific bands for the Scaffold_241699. It is further tested in new individuals.*

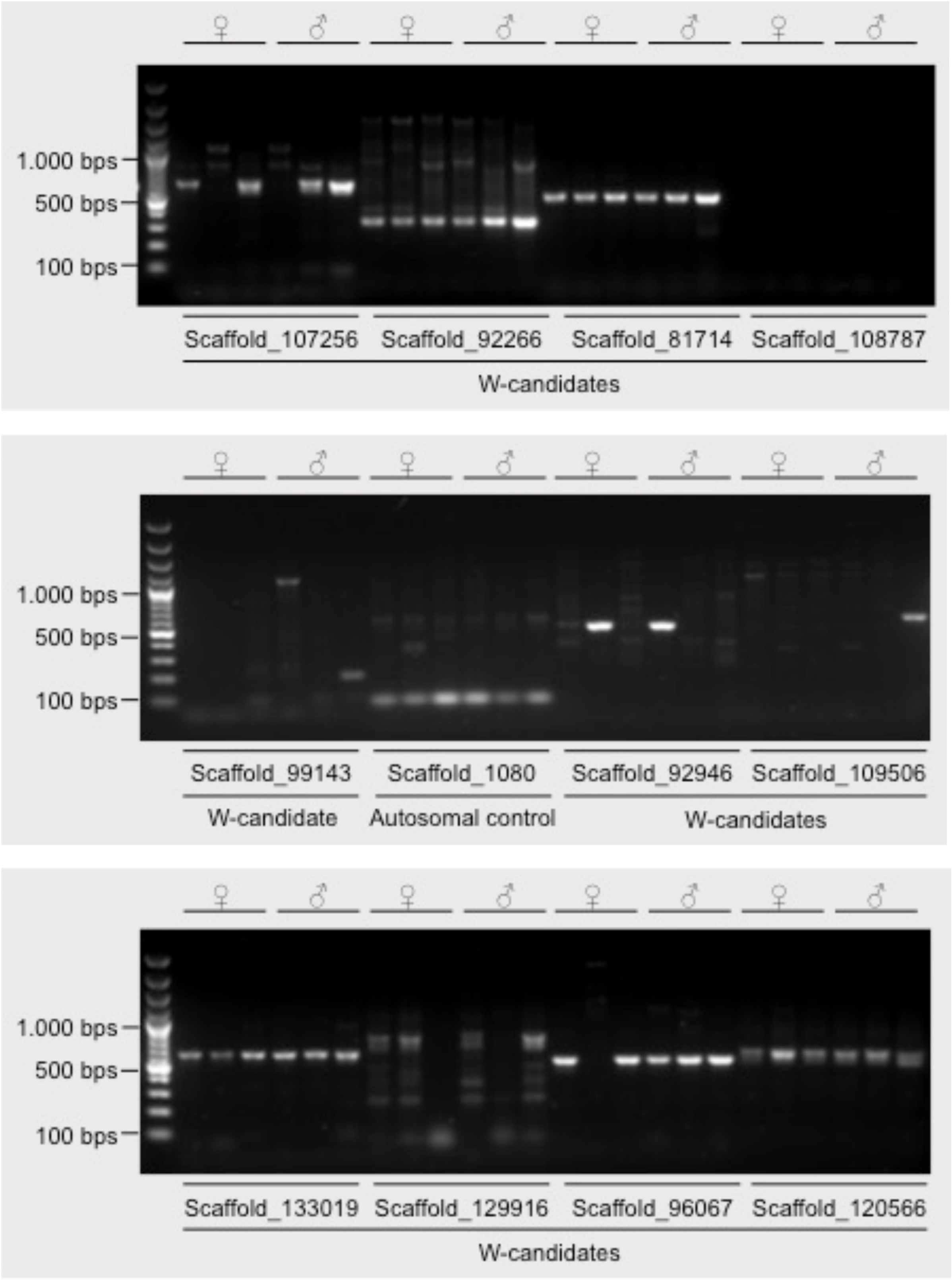
♀ ♂ ♀ ♂ ♀ ♂ ♀ ♂
1.000 bps
500 bps
100 bps
Scaffold_107256
Scaffold_92266
Scaffold_81714
Scaffold_108787
W-candidates
♀ ♂ ♀ ♂ ♀ ♂ ♀ ♂
1.000 bps
500 bps
100 bps
Scaffold_99143
Scaffold_1080
Scaffold_92946
Scaffold_109506
W-candidate
Autosomal control
W-candidates
♀ ♂ ♀ ♂ ♀ ♂ ♀ ♂
1.000 bps
500 bps
100 bps
Scaffold_133019
Scaffold_129916
Scaffold_96067
Scaffold_120566
W-candidates

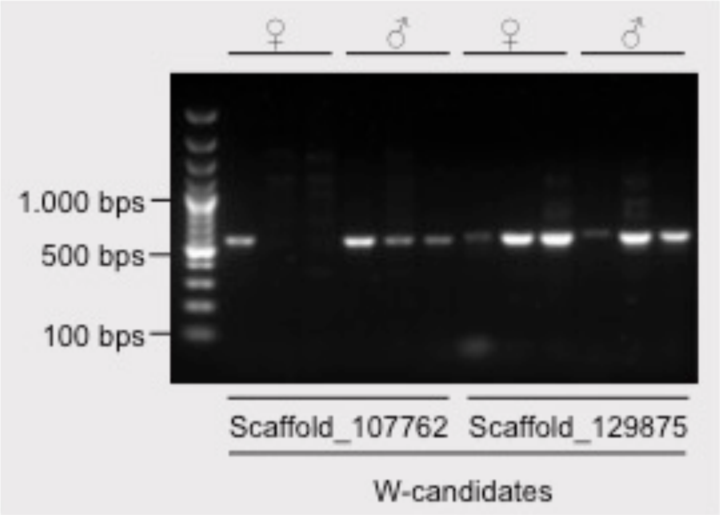

Additional test of Scaffold_241699

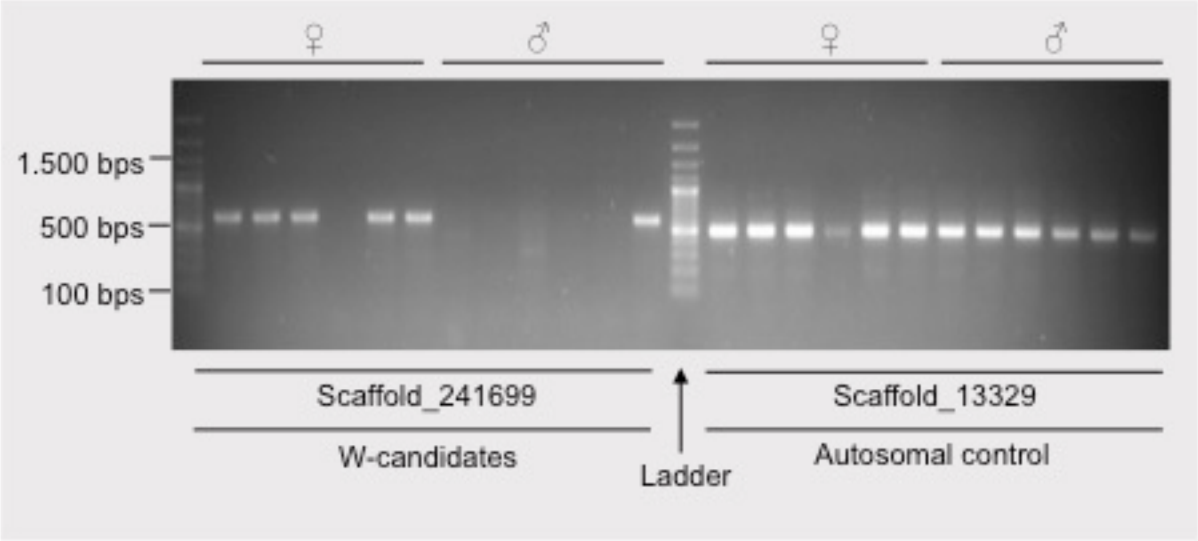

Supplementary Figure 5: Testing the female specificity of the W-candidates in (A) ***C. ohridella.*** **(B)** ***N. degeerella*****. (C)** ***T. sylvina*****.** Amplification patterns of genomic DNA from three females and three males are shown for each W-candidate, as well as for two autosomal control genes. In *C. ohridella*, female-specific PCR markers are indicated by an asterisk. In each of the two non-ditrysian species, one spurious female-specific candidate (indicated by a question mark) was further tested on four other males and females for *N. degeerella*, and three other males and females for *T. sylvina*. The molecular size marker is indicated on the side (100 bp ladder). Primer sequences and expected length of the PCR products are provided in Supplementary Table 7.

Supplementary Table 1: Library information.

Species	*De novo* sequencing library ID	Tissue	Sex	Number of individuals	Adapter	Insert Size	Model	Type	Number of reads
N. degeerella	37850	whole body	female	1	Default:TrueSeq 3:TTAGGC	604	HiSeqV4	PE125	55,674,308
N. degeerella	37851	whole body	female	1	Default:TrueSeq 14:AGTTCC	506	HiSeqV4	PE125	71,599,626
N. degeerella	37852	whole body	male	1	Default:TrueSeq 16:CCGTCC	502	HiSeqV4	PE125	59,822,239
N. degeerella	37853	whole body	male	1	Default:TrueSeq 6:GCCAAT	474	HiSeqV4	PE125	62,063,497
N. degeerella	52195	whole body	male	9	NEBNext Dual 707:CTGAAGCT + 503:CCTATCCT	674	HiSeqV4	PE125	191,194,381
C. ohridella	44575	whole body	male	1	Default:TrueSeq 19:GTGAAA	434	HiSeqV4	PE125	48,865,107
C. ohridella	44576	whole body	male	10	Default:TrueSeq 8:ACTTGA	511	HiSeqV4	PE125	76,533,114
C. ohridella	44577	whole body	female	1	Default:TrueSeq 5:ACAGTG	532	HiSeqV4	PE125	60,213,476
C. ohridella	44578	whole body	female	10	Default:TrueSeq 11:GGCTAC	477	HiSeqV4	PE125	66,373,223
T. sylvina	44579	head	male	1	Default:TrueSeq 5:ACAGTG	465	HiSeqV4	PE125	127,751,074
T. sylvina	44580	head	female	1	Default:TrueSeq 11:GGCTAC	445	HiSeqV4	PE125	109,822,118
T. sylvina	44580 (resequenced)	head	female	1	Default:TrueSeq 11:GGCTAC	445	HiSeqV4	PE125	176,710,674
T. sylvina	Publicly available	-	male	1	-	-	HiSeq2000	PE100	-
L. lunatus	Publicly available	-	female	several	-	-	HiSeq2000	PE100	-

Species	*De novo* sequencing library ID	SRA accession number	BioSample accession number	Study accession number	Bioproject accession number
N. degeerella	37850	SRR5626451	SAMN07172865	SRP108218	PRJNA388200
N. degeerella	37851	SRR5626450	SAMN07172866	SRP108218	PRJNA388200
N. degeerella	37852	SRR5626449	SAMN07172867	SRP108218	PRJNA388200
N. degeerella	37853	SRR5626448	SAMN07172868	SRP108218	PRJNA388200
N. degeerella	52195	SRR5884493	SAMN07428209	SRP108218	PRJNA388200
C. ohridella	44575	SRR5626455	SAMN07172869	SRP108218	PRJNA388200
C. ohridella	44576	SRR5626454	SAMN07172870	SRP108218	PRJNA388200
C. ohridella	44577	SRR5626453	SAMN07172871	SRP108218	PRJNA388200
C. ohridella	44578	SRR5626452	SAMN07172872	SRP108218	PRJNA388200
T. sylvina	44579	SRR5626447	SAMN07172873	SRP108218	PRJNA388200
T. sylvina	44580	SRR5626446	SAMN07172874	SRP108218	PRJNA388200
T. sylvina	44580 (resequenced)	SRR5884531	SAMN07172874	SRP108218	PRJNA388200
T. sylvina	Publicly available	SRR1190481	SAMN02688767	SRP040078	PRJNA241175
L. lunatus	Publicly available	SRR947083 to SRR947088	SAMN02211182	SRP028130	PRJNA203303

Supplementary Table 2: Genome assembly statistics for *C. ohridella*, *N. degeerella*, and *T. sylvina*
Only scaffolds longer than 200 bp were considered.

Species: *C. ohridella*					
Starting library: 44577					
	Sequence #	Total length	Max length	N50	N90
Soap de novo	685,168	478,576,601	94,034	3,832	167
Gap Closer	685,168	453,762,297	89,979	3,365	167
Filter > 200bp	262,071	393,510,795	89,979	4,305	494
Sspace	181,756	394,232,181	114,100	6,133	958
Cap3	152,025	388,643,033	114,100	6,277	1,175

Species: *N. degeerella*					
Starting library: 37850					
	Sequence #	Total length	Max length	N50	N90
Soap de novo	913,972	693,820,722	79,562	4,692	165
Gap Closer	913,972	594,602,582	70,036	3,435	135
Filter > 200bp	284,771	512,913,114	70,036	4,285	728
Sspace	221,391	521,588,503	70,036	5,219	1,072
Cap3	194,957	517,135,461	70,036	5,350	1,224

Species: *T. sylvina*					
Starting library: 44580					
	Sequence #	Total length	Max length	N50	N90
Soap de novo	5,309,483	2,192,689,788	26,951	832	129
Gap Closer	5,309,483	2,196,238,786	27,331	831	129
Filter > 200bp	2,537,614	1,821,941,495	27,331	1,064	276
Sspace	2,353,920	1,821,463,072	243,662	1,184	286
Cap3	-	-	-	-	-

Supplementary Table 3: Chi-squared test of the number of Z-linked and autosomal genes in pairwise comparisons with *B. mori*.

Occurrence of each category is based on Supplementary Data 3-6. Only scaffolds longer than 1,500 bp with coverage above 5 and below the 99.5th percentile in the grouped male sample were considered. *P-values* are given for the Chi-squared test and the Fisher's exact test (two-tailed). Unclassified genes are tagged as "NA".

A. Lenient classification

	Z-linked	Autosomal	TOTAL
B. mori	363	8,760	**9,123**
C. ohridella (NA=18)	484	8,621	**9,105**
TOTAL	**847**	**17,381**	

Chi2: *P* < 0.0001; Fisher: "unable to compute *P*, too large values"

	Z-linked	Autosomal	TOTAL
B. mori	342	8,017	**8,359**
N. degeerella (NA=22)	300	8,037	**8,337**
TOTAL	**642**	**16,054**	

Chi2: *P* = 0.0976; Fisher: "unable to compute *P*, too large values"

	Z-linked	Autosomal	TOTAL
B. mori	156	4,897	**5,053**
T. sylvina (NA=167)	138	4,748	**4,886**
TOTAL	**294**	**9,645**	

Chi2: *P* = 0.4393; Fisher: *P* = 0.4422

B. Stringent classification

	Z-linked	Autosomal	TOTAL
B. mori	363	8,760	**9,123**
C. ohridella (NA=720)	474	7,929	**8,403**
TOTAL	**837**	**16,689**	

Chi2: *P* < 0.0001; Fisher: "unable to compute *P*, too large values"

	Z-linked	Autosomal	TOTAL
B. mori	342	8,017	**8,359**
N. degeerella (NA=788)	314	7,257	**7,571**
TOTAL	**656**	**15,274**	

Chi2: *P* = 0.8590; Fisher: "unable to compute *P*, too large values"

	Z-linked	Autosomal	TOTAL
B. mori	156	4,897	**5,053**
T. sylvina (NA=545)	78	4,430	**4,508**
TOTAL	**234**	**9,327**	

Chi2: *P* < 0.0001; Fisher: *P* < 0.0001

Supplementary Table 4: Correspondence between Z-linked and autosomal genes in pairwise comparisons with *B. mori*.

Occurrence of each category is based on Supplementary Data 3-6. Only scaffolds longer than 1,500 bp with coverage above 5 and below the 99.5th percentile in the grouped male sample were considered. Based on the Silkworm Genome Database (v2.0, http://silkworm.genomics.org.cn), 654 Z-linked genes are annotated in *B. mori*. Unclassified genes are tagged as "NA".

A. Lenient classification

		B. mori	
		Z-linked (Chr. 1)	Autosomal
C. ohridella	Z-linked	301	183
	Autosomal	56	8,565
	NA	6	12

		B. mori	
		Z-linked (Chr. 1)	Autosomal
N. degeerella	Z-linked	280	20
	Autosomal	49	7,988
	NA	13	9

		B. mori	
		Z-linked (Chr. 1)	Autosomal
T. sylvina	Z-linked	93	45
	Autosomal	36	4,712
	NA	27	140

B. Stringent classification

		B. mori	
		Z-linked (Chr. 1)	Autosomal
C. ohridella	Z-linked	298	176
	Autosomal	45	7,884
	NA	20	700

		B. mori	
		Z-linked (Chr. 1)	Autosomal
N. degeerella	Z-linked	290	24
	Autosomal	42	7,215
	NA	10	778

		B. mori	
		Z-linked (Chr. 1)	Autosomal
T. sylvina	Z-linked	57	21
	Autosomal	23	4,407
	NA	76	469

Supplementary Table 5: Chi-squared test of the number of genes moving onto and off the Z chromosome across all species.
Occurrence of each category is based on Supplementary Data 1. *P-values* are given for the Chi-squared test and the Fisher's exact test (two-tailed).

A. Lenient classification

	Onto-Z	Off-Z	TOTAL
Ditrysia	5	1	**6**
N. degeerella + B. mori	13	11	**24**
TOTAL	**18**	**12**	

Chi2: *P* = 0.1921; Fisher: *P* = 0.3575

	Onto-Z	Off-Z	TOTAL
C. ohridella	91	5	**96**
N. degeerella + B. mori	13	11	**24**
TOTAL	**104**	**16**	

Chi2: *P* < 0.0001; Fisher: *P* < 0.0001

B. Stringent classification

	Onto-Z	Off-Z	TOTAL
Ditrysia	2	1	**3**
N. degeerella + B. mori	9	7	**16**
TOTAL	**11**	**8**	

Chi2: *P* = 0.7374; Fisher: *P* = 1

	Onto-Z	Off-Z	TOTAL
C. ohridella	73	2	**75**
N. degeerella + B. mori	9	7	**16**
TOTAL	**82**	**9**	

Chi2: *P* < 0.0001; Fisher: *P* < 0.0001

Supplementary Table 6: Chi-squared test of the number of genes moving onto and off the Z chromosome across all species, obtained by applying a minimal scaffold length of 500 bp.
Occurrence of each category is based on Supplementary Data 1. *P-values* are given for the Chi-squared test and the Fisher's exact test (two-tailed).

A. Lenient classification

	Onto-Z	Off-Z	TOTAL
Ditrysia	7	1	**8**
N. degeerella + B. mori	17	14	**31**
TOTAL	**24**	**15**	

Chi2: *P* = 0.0905; Fisher: *P* = 0.1214

	Onto-Z	Off-Z	TOTAL
C. ohridella	123	13	**136**
N. degeerella + B. mori	17	14	**31**
TOTAL	**140**	**27**	

Chi2: *P* < 0.0001; Fisher: *P* < 0.0001

Stringent classification

	Onto-Z	Off-Z	TOTAL
Ditrysia	5	1	**6**
N. degeerella + B. mori	10	3	**13**
TOTAL	**15**	**4**	

Chi2: *P* = 0.7500; Fisher: *P* = 1

	Onto-Z	Off-Z	TOTAL
C. ohridella	102	5	**107**
N. degeerella + B. mori	10	3	**13**
TOTAL	**112**	**8**	

Chi2: *P* = 0.0120; Fisher: *P* = 0.0409

Supplementary Table 7: Primer sequences, expected length of PCR products and validation of the targets as W-specific sequences.

Species: *C. ohridella*				
**Target**	**Primer**	**Sequence (5' to 3')**	**Expected Product Size (bp)**	**Validation**
Contig_9175 (Autosomal control)	Forward<br>Reverse	TGTTGCTCTCAGCCTCTGTG<br>ACGATGTCTGGTGTCACCAC	651	N.A.
Scaffold_3409 (Autosomal control)	Forward<br>Reverse	GCGATGTACGTAGCCATCCA<br>CGATACCGGGGTACATGGTG	538	N.A.
Scaffold_49092	Forward<br>Reverse	CCTACTGCAGGCCATATGGG<br>TCGACAAACTGCCGAGTTCA	600	Yes
Scaffold_60890	Forward<br>Reverse	GTCCTCAATATGCCTCGCGA<br>GCACCCTGAAACGTTGAACC	586	Yes
Scaffold_61507	Forward<br>Reverse	GGTAGGCGAAGTAAGGTCGG<br>CCCTTGATCTGCCCTTGACA	584	Yes
Scaffold_63777	Forward<br>Reverse	GCGATGAGAGGACTTCCAGT<br>GCCAGGACTTGAACATCGGA	539	No
Scaffold_65032	Forward<br>Reverse	TTGCATTGTTGGGGCGAAAG<br>GCCTCGCAGTCCTTTTAGCT	536	Yes
Scaffold_65287	Forward<br>Reverse	ACCCTCTAGGCTTCATTGCG<br>GGGAAAGCCATGATGGACCT	524	Yes
Scaffold_65672	Forward<br>Reverse	GGCGAAGATATCAACTGCACA<br>AGGTCACAGTTGGAATACGCA	405	No
Scaffold_68206	Forward<br>Reverse	GGATCATGACGAAGAATCACGC<br>TGACGCGTTGTTACACATTGAC	531	Yes
Scaffold_68940	Forward<br>Reverse	TCTGGGATAGTAGGGTGCTGT<br>GGAGACCTACCTACCCCCAT	502	No
Scaffold_72377	Forward<br>Reverse	ATGGGGGTTTTCCTGGAACTC<br>CTATGAGCCCATTTTTCGCCG	501	No

Species: *N. degeerella*				
**Target**	**Primer**	**Sequence (5' to 3')**	**Expected Product Size (bp)**	**Validation**
Scaffold_1463 (Autosomal control)	Forward<br>Reverse	CGATACCGGGGTACATGGTG<br>CCCGCCATGTATGTAGCCAT	541	N.A.
Scaffold_17189 (Autosomal control)	Forward<br>Reverse	TCCAAATGACCCACTCGCTC<br>ATCTGCTCCTCGACCTCCTT	592	N.A.
Scaffold_101347#1	Forward<br>Reverse	GCCTTATGGCAACAAGTTCG<br>TTGTTTACTCCGTTTTAAAGTCCTG	102	No
Scaffold_101347#2	Forward<br>Reverse	GCCTTATGGCAACAAGTTCG<br>AACAGTAAAACGAGACGAAGAAAA	132	No
Scaffold_102546	Forward<br>Reverse	GACTGTACGTCAACAAAGACACT<br>GGGATTATTGGATGTAGGAAAATTACA	597	No

Scaffold_115426	Forward<br>Reverse	AGATCGATTAGGCGGGTTTT<br>TGCCTTATTTTGATGCCACA	235	No
Scaffold_132633	Forward<br>Reverse	AAATGGCTGGAAGGAAAGGT<br>CCAATGTAAAAGGGGACGTG	245	No
Scaffold_137761	Forward<br>Reverse	ATAGCTGCGACAAGGACGTT<br>TGTTGGTCAAACCGAACTGA	165	No
Scaffold_147560	Forward<br>Reverse	CGTGCGCCTCTTGTTTTATT<br>AATAGCTTTATTTTGGTATAAGCACA	153	No
Scaffold_153063#1	Forward<br>Reverse	TGCAATATGCAGATGACCTTG<br>TGTTGGACATGCTTCTTGGA	172	No
Scaffold_153063#2	Forward<br>Reverse	ACTCGGCGTAGGTTTTGCAT<br>TTCTGATGACAACGGCAAGA	152	No
Scaffold_160976	Forward<br>Reverse	AGCATCAAATGCTCGATGAA<br>CAATGAGACTCACCTGGATGG	207	No
Scaffold_161417	Forward<br>Reverse	GGGCCTATATGCCACAACAA<br>AAACGAGCCTGAAATTGACC	220	No
Scaffold_165896	Forward<br>Reverse	CGCTCACACTGACAGGGATA<br>TCGATTCGTGATCATGTCGT	230	No
Scaffold_166636	Forward<br>Reverse	TCGACCTCGGAACTCAAATG<br>ACAGAGAAGAGCCGGCAATA	170	No
Scaffold_170525	Forward<br>Reverse	CAGTGTCGCAATACATGAAACA<br>GACAGGACGTAATGTCACTTTG	229	No
Scaffold_177914	Forward<br>Reverse	TGGATGATAAAGCGAAAACG<br>GCTTGGCTGGAGTCCAAAAT	212	No
Scaffold_178674	Forward<br>Reverse	TGCGTCGGGGTTAAAAATAG<br>GAAAAGAGGAACCGCACATC	198	No
Scaffold_178933	Forward<br>Reverse	GCACCGCATCGTAGAATAGA<br>ATGGACAACAACCACGAAGC	169	No
Scaffold_185022	Forward<br>Reverse	TGAACGACCATGAAATACCG<br>TTGTGAATTCAGCCAAGCAG	154	No
Scaffold_192322	Forward<br>Reverse	GCATGAAACACGTCTCAACG<br>TTTACACGCTTGTGATTGTCG	181	No
Scaffold_193918	Forward<br>Reverse	TTTGGCGGAAAATATGAAGG<br>GAACAGTATGTGTTGGGTGAAAA	187	No
Scaffold_199997	Forward<br>Reverse	TTTTCATAATCCGCGTTTCC<br>ACTAAACGCCGCAGACTGAT	184	No
Scaffold_201975	Forward<br>Reverse	AAGCTGAAGAAGACGCAGATG<br>TGAATTAGGAAAAACCAACAGGA	152	No
Scaffold_203418	Forward<br>Reverse	AAAAGAAAATCGCTGTTATGATTTTT<br>CGTCATCATCAAAAAGAATTTGG	152	No
Scaffold_205965	Forward<br>Reverse	TCGCCAATAAAGCAGAGTGA<br>GAATTATTTACTGGGCGGAGAA	100	No
Scaffold_208476	Forward<br>Reverse	GGTCCCGTTTTTACCCTTTG<br>CGCTATTTCGTCGAGAATGTT	190	No
Scaffold_213938	Forward<br>Reverse	AAATGTGAAATACAGCTTTGCTTG<br>AAAAACCGTGGGCTTGCTA	153	No

Scaffold_215252	Forward<br>Reverse	GCCCAACTCAACTCACGTTAT<br>AACAGGGTCCCGTTTTTACC	121	No
Scaffold_216293	Forward<br>Reverse	TGAAAAGTGAATACCGCTTCAA<br>AACTTGGCACGACTTCAAAAA	177	No
Scaffold_218862	Forward<br>Reverse	CGTCGAATTGAGAACCTCCT<br>TTTTCATTCATTCATTCATTCATT	189	No
Scaffold_220972	Forward<br>Reverse	CAGAGCCAACCCAATACTCG<br>TGGTCGAAGGTTTTAGAATAGTTG	150	No

Species: *T. sylvina*

**Target**	**Primer**	**Sequence (5' to 3')**	**Expected Product Size (bp)**	**Validation**
Scaffold_1080 (Autosomal control)	Forward<br>Reverse	CTCTCCGATACCGCTGTGAC<br>GGATAGCCATCGACCTGTGG	594	N.A.
Scaffold_13329 (Autosomal control)	Forward<br>Reverse	GAAGTGGAGACGTGGGAAGG<br>AACAACTGGGCAAAGGGACA	501	N.A.
Scaffold_57334	Forward<br>Reverse	TCCATTGGTTTTGTGCCCCT<br>GCTAATAGAAACGGGGCTCGA	503	No
Scaffold_81714	Forward<br>Reverse	CAGAGTCAACCCTGCGTCTT<br>AGCTGTCACGACTGGTCATG	513	No
Scaffold_83004	Forward<br>Reverse	TCGTGGACCAATTTTTCGGC<br>AATTCCAGTACTGTGCGGCA	501	No
Scaffold_92266	Forward<br>Reverse	GAAAACCGAGGACAACACGC<br>GAAAACCGAGGACAACACGC	524	No
Scaffold_92946	Forward<br>Reverse	TCCAGACGTCTTGTACACGC<br>CATCGCCATCATACCACCCA	510	No
Scaffold_96067	Forward<br>Reverse	CCACTGCGGTGTACTTAGCA<br>ATTTCGTGGCGGACTCCAAA	547	No
Scaffold_96075	Forward<br>Reverse	GACAGAAGCTAGGATCCCGC<br>TCGTGTGCGAGGATCCAAAA	549	No
Scaffold_99143	Forward<br>Reverse	TGTTCCAGTAGTGCGGCATT<br>TCACCGAATCACACTGCCAA	523	No
Scaffold_107256	Forward<br>Reverse	ACCACGCACTACTTTGGAGG<br>CCTGAAGTCGAGAAACGCGA	553	No
Scaffold_107762	Forward<br>Reverse	GTTCTTCGGGCGGATCTACC<br>TCACCAACGGATGCTTTGAGT	562	No
Scaffold_108787	Forward<br>Reverse	AGTTATGTACACATTCTTCCTAGGTGT<br>ACAAATCGATGCGTCCCTCT	402	No
Scaffold_109506	Forward<br>Reverse	TGCTGTGGCGGCTTAACTTA<br>TTTGGTGGGAAGATGGCCAG	570	No
Scaffold_117957	Forward<br>Reverse	TCCCAAAGGTGAGAGGGACT<br>AGGACGGGGAGTTAAACCCT	515	No
Scaffold_120566	Forward<br>Reverse	TTCCCCATGCTACGTTCCTG<br>CCACACCCACTGCAATCTCT	554	No
Scaffold_122749	Forward	GCGCCACCAAAGAAACGAAA	539	No

	Reverse	GCAAGCCCATACGAGTGTGT		
Scaffold_123444	Forward	GTAGGCAGCAAACCAGGACT	572	No
	Reverse	ACTCTGCAACTGTCTCTGCC		
Scaffold_127132	Forward	CTACTGCTGCCCTGGAACAA	581	No
	Reverse	GAATCAACCAGGCCGAATGC		
Scaffold_129875	Forward	AGGGCCTGATTTCACAATGGA	511	No
	Reverse	ACCCTGCATTTGTAGGCGAT		
Scaffold_129916	Forward	GGATGAATAGTGGCCTGCCT	600	No
	Reverse	TAGCCACAGGTTAAGCCAGC		
Scaffold_130174	Forward	CGGTATCGGTGGCTCTCAAG	599	No
	Reverse	TCCGAATTGAGCGACGTACA		
Scaffold_133019	Forward	TGTACGTCGCTATCACGCAG	595	No
	Reverse	TAGAAGTTGGCACCGCGTAG		
Scaffold_137663	Forward	CTTCACGGACGATGTGAGCT	505	No
	Reverse	GCGCTCGATCTACCTTTCCA		
Scaffold_241699	Forward	CACAATGGTTGCAATGGCGA	528	No
	Reverse	CTGGCTGCTAAAGGGACCAA		

Supplementary References

1. Regier, J. C. *et al.* A Large-Scale, Higher-Level, Molecular Phylogenetic Study of the Insect Order Lepidoptera (Moths and Butterflies). *PLoS One* **8,** e58568 (2013).
