# Supplementary material for: The deep conservation of the Lepidoptera Z chromosome suggests a non-canonical origin of the W
Source: Nat Commun. 2017 Nov 14;8:1486. doi: 10.1038/s41467-017-01663-5 (PMC5684275; doi:10.1038/s41467-017-01663-5)
Supplement: Supplementary file 3 — Description of Additional Supplementary Files [file 41467_2017_1663_MOESM3_ESM.pdf]

File Name: Supplementary Data 1
Description: Scaffold classification and homology with *B. mori* chromosomes in *C. ohridella*, *N. degeerella*, and *T. sylvina*.

File Name: Supplementary Data 2
Description: *De novo* assembly (Example of *N. degeerella*).

File Name: Supplementary Data 3
Description: Coverage values for each library and after keeping only uniquely mapped reads in *C. ohridella*.

File Name: Supplementary Data 4
Description: Coverage values for each library and after keeping only uniquely mapped reads in *N. degeerella*.

File Name: Supplementary Data 5
Description: Coverage values for each library and after keeping only uniquely mapped reads in *T. sylvina*.

File Name: Supplementary Data 6
Description: Coverage values for each library and after keeping only uniquely mapped reads in *L. lunatus*.

File Name: Supplementary Data 7
Description: Bash script for reference mapping.

File Name: Supplementary Data 8
Description: DNA coverage estimation.

File Name: Supplementary Data 9
Description: CDS *Bombyx mori*.

File Name: Supplementary Data 10
Description: Chromosomal location of *Bombyx mori* CDS.

File Name: Supplementary Data 11
Description: Female and male coverage values of scaffolds assigned to *B. mori* chromosomes in *C. ohridella*.

File Name: Supplementary Data 12
Description: Female and male coverage values of scaffolds assigned to *B. mori* chromosomes in *N. degeerella*.

File Name: Supplementary Data 13
Description: Female and male coverage values of scaffolds assigned to *B. mori* chromosomes in *T. sylvina*.

File Name: Supplementary Data 14
Description: Female and male coverage values of scaffolds assigned to *B. mori* chromosomes in *L. lunatus*.

File Name: Supplementary Data 15
Description: BLAT mapping of scaffolds onto *B. mori* genome.

File Name: Supplementary Data 16
Description: R code used to detect *in silico* Z-linked scaffolds.

File Name: Supplementary Data 17
Description: W-candidates in fasta format for *C. ohridella*.

File Name: Supplementary Data 18
Description: W-candidates in fasta format for *N. degeerella*.

File Name: Supplementary Data 19
Description: W-candidates in fasta format for *T. sylvina*.

File Name: Supplementary Data 20
Description: R code used to detect *in silico* W-candidates.

File Name: Supplementary Data 21
Description: Scaffold classification in *T. sylvina*, before adding the publicly available individual.

File Name: Supplementary Data 22
Description: R code used to identify the sex of the publicly available *T. sylvina* individual.
